# Sponge Tents, Preparation of

**Published:** 1875-07

**Authors:** 


Preparation of Sponge-Tents.—Having for the past two years met with *decided success* in the use of sponge-tents, it may be of interest to know how I prepare them. The formula I adopt is, first, to make a saturated infusion of hydrastis canadensis, to which is added enough of gum accacia to make the infusion of the proper consistence; then, for every one dozen tents to be prepared, I add—

℞.—Acidi carbolici, . . . . . . . . gr. xxiv.

Ol. Caryophyli, . . . . . . . . gtt. xij.

*Misce bene,* and add to the infusion. Then proceed as usual in the manufacturing of tents—insert the wire, wind, trim when dry, and coat with gelatin.

The propriety of using the above will be apparent at once. Tents thus made are the best I have ever used.—*Paul Boyce, M.D. Va. Medical Journal.*

